# Supplementary material for: Patterns and predictors of outcome monitoring amongst link workers: Learnings from the National Social Prescribing Link Worker Survey 2025
Source: PLoS One. 2026 Apr 29;21(4):e0346234. doi: 10.1371/journal.pone.0346234 (PMC13127906; doi:10.1371/journal.pone.0346234)
Supplement: S1 File — (DOCX) [file pone.0346234.s001.docx]

**Supplementary methods**

Variables used in analyses

| Question | Response Options |
| --- | --- |
| Demographics |  |
| If your service is set up to support a specific cohort of patients and/or clients, please select from the following.* (Please select all that apply. If your service is set up to support a long-term condition that is not listed, please select ‘Other’ and specify.) | ☐ Children & Young People ☐ Over 65s / Older People ☐ Frailty ☐ Mental Health ☐ Learning Disabilities ☐ Employment Support ☐ Cancer ☐ Dementia ☐ Chronic Fatigue ☐ Musculoskeletal (MSK) conditions ☐ LGBTQ+ ☐ Refugee & Asylum Seekers ☐ Other |
| What category of organisations do you make the most referrals to? (Select up to 5) | ☐ Age‑related activities (Youth groups, Age UK, etc.) ☐ Arts & Culture ☐ Faith‑based activities ☐ Green or Nature‑based activities ☐ Healthcare services (secondary care, specialists, back to referrer) ☐ Mental health support services ☐ Heritage activities ☐ Carers support/services ☐ Domestic abuse support/services ☐ Employment support/services (e.g., Work‑Well, DWP) ☐ Financial support/services ☐ Housing support/services ☐ Legal/Welfare advice services ☐ Social support/services ☐ Physical activity sector activities ☐ Other |
| RQ1 |  |
| Do you record and/or monitor your onward referrals in the following ways? | (very often, often, sometimes, rarely, never, not applicable) ○ Record and/or monitor patient outcomes after the referral ○ Record and/or monitor impact on services after the referral |
| Overall, how often do you record patient and/or client outcome measures (e.g., ONS4)? | ○ Very often ○ Often ○ Sometimes ○ Rarely ○ Never |
| Do you capture social prescribing outcomes through any of the following qualitative methods? (Select all that apply) | ☐ Patient and/or client experience surveys ☐ Patient and/or client stories ☐ Career feedback ☐ Referrer feedback e.g. feedback from VCSE organisations ☐ Case studies ☐ None ☐ Other |
| To what extent do you agree with the following statements? | (Strongly disagree, disagree, neither agree not disagree, agree, strongly agree) - I have had training on the local clinical system (i.e. EMIS Web or TPP SystmOne) - I am aware of the Social Prescribing Information Standard - I am familiar with SNOMED codes - I feel confident using the local clinical system to add SNOMED codes to patient and/or client records - I am able to access NHS patient and/or client records |
| Are you a SPLW Team Lead, SPLW Manager, and/or Senior SPLW? | ○ Yes ○ No |
| Which forms of supervision do you receive in your role? *Please select all that apply.* | ☐ I have a first point of contact for general advice and support ☐ I have a named GP supervisor and/or access to a GP for raising patient and/or client concerns and safeguarding issues ☐ I have a clinical supervisor who is not a GP and not my named day-to-day line manager ☐ I have a non-clinical workplace supervisor who is not a GP ☐ I receive workplace supervision from another Social Prescribing Link Worker ☐ I receive workplace supervision from a Social Prescribing Manager and/or Lead ☐ I pay for my own supervision ☐ Peer support sessions arranged within your ICS ☐ Peer support sessions arranged within your PCN and/or organisation ☐ Peer support sessions arranged across a number of PCNs ☐ Peer support on a one-to-one basis ☐ Peer support sessions arranged around a specialism or interest (e.g Children and Young People) ☐ Peer support with other personalised care roles ☐ I have not received any peer support ☐ I have not received any workplace supervision |
| Approximately what is the amount you have for a training budget per year as a social prescribing link worker? | ○ Less than £500 ○ £501–£1500 ○ £1501–£3000 ○ More than £3000 ○ I don’t know ○ I don’t have access to a training budget |
| RQ2 |  |
| What is your age group? | ○ Under 18 ○ 18–24 ○ 25–34 ○ 35–44 ○ 45–54 ○ 55–64 ○ 65+ ○ Prefer not to say |
| What is your gender identity? | ○ Man ○ Woman ○ Non-binary ○ Prefer not to say ○ Not listed, please self-describe |
| What is your ethnic group? | ○ Asian or Asian British (including Indian, Pakistani, Bangladeshi, Chinese) ○ Black or Black British (includes African, Caribbean) ○ Mixed or Multiple Ethnic Groups (includes White and Black Caribbean, White and Black African, White and Asian) ○ White (includes British, Northern Irish, Irish, Gypsy, Roma, Traveller) ○ Prefer not to say ○ Not listed or prefer to self-define |
| Do you identify as disabled? | ○ Yes ○ No ○ Prefer not to say |
| What is the highest level of training and/or education you had received before becoming an SPLW? | ○ Level 1–2 (GCSE, intermediate apprenticeship) ○ Level 3–4 (A-level, BTEC nationals, Advanced apprenticeship) ○ Level 6 (Undergraduate degree, foundation degree, higher apprenticeship) ○ Level 7 (Master’s degree) ○ Level 8 (Doctorate PhD) ○ Not Applicable |
| Where did you work prior to becoming an SPLW? (select all that apply) | ☐ NHS clinical role ☐ NHS non-clinical role ☐ Social Care ☐ Local Authority ☐ VCSE sector ☐ Education ☐ Private sector ☐ in a volunteering role ☐ This is my first professional role ☐ Other |
| Have you considered or might you consider resigning from your role in the next year? | ○ Yes ○ No |
| Which region do you work in? | ○ North East & Yorkshire ○ North West ○ Midlands ○ East of England ○ South East ○ South West ○ London |
| Where are you based in your current role? (select all that apply) | ☐ GP Surgery and/or PCN ☐ Secondary or acute care setting ☐ Hospital Discharge ☐ VCSE organisation ☐ Community venue ☐ Children's Centre and/or Family Hub ☐ Remote and/or Work from Home ☐ Other ☐ Not Applicable |
| How is your post funded? | ☐ ARRS (Additional Roles Reimbursement Scheme) ☐ ICB (Integrated Care Board) ☐ Local Authority ☐ Voluntary Sector Grant and/or Commission (National Lottery, Foundation, Community Grant etc) ☐ I don’t know ☐ Other |
| What is your approximate annual caseload? | ○ 0–50 ○ 51–100 ○ 101–150 ○ 151–200 ○ 201–250 ○ 251–300 ○ 300+ |
| With whom are the outcomes shared? (Select all that apply) | ☐ The referrer ☐ My employer / organisation / manager ☐ ICB ☐ Local Authority ☐ The reports are publicly shared (e.g. via a website) ☐ PCN ☐ Clinical Director ☐ A Local Social Prescribing Network ☐ Other |
| Has outcomes data been used to influence finance or investment decisions? | ○ Yes ○ No ○ I do not know |
| RQ3 |  |
| To what extent do you agree or disagree with the following statements? | (Strongly disagree, disagree, neither agree not disagree, agree, strongly agree) - I feel that my work has a positive impact on the people I support |
| Based on your observations, how has social prescribing influenced patients and/or clients in the areas below? | (Strong positive impact |
| How effective do you feel the following are in supporting your patients/clients? | (Very effective |
| RQ4 |  |
| On average, how regularly do you collect outcomes with a patient and/or client? | ○ Every 6 weeks ○ Every 12 weeks ○ Every 6 months ○ At every interaction ○ At the beginning and end of working with a patient and/or client ○ I do not regularly collect patient and/or client outcomes ○ Other |
| With whom are the outcomes shared? (Select all that apply) | ☐ The referrer ☐ My employer / organisation / manager ☐ ICB ☐ Local Authority ☐ The reports are publicly shared (e.g. via a website) ☐ PCN ☐ Clinical Director ☐ A Local Social Prescribing Network ☐ Other |
| Which tools do you use to measure social prescribing outcomes? (select all that apply) | ☐ ONS4 ☐ Wellbeing Star ☐ WEWBWBS ☐ SWEMWBS ☐ MYCaW ☐ PAM ☐ Own / In-house ☐ None ☐ Other |
| What IT system do you use to track and/or monitor your referrals? | ☐ Clinical system (EMIS, System One) ☐ Joy ☐ Elemental ☐ Social RX ☐ Excel spreadsheet ☐ In-house CRM ☐ I do not have an IT system to track referrals ☐ Other |
| RQ5 |  |
| To what extent do you agree with the following? | (Strongly disagree, disagree, neither agree not disagree, agree, strongly agree) These skills are important for my role: - Understanding of outcome measurement tools - Confidence in using data systems (including data capture and reporting) - Understanding the benefits of a variety of activities for an individuals health and wellbeing |
| What do you feel you need to be able to consistently capture client outcomes more regularly? (Select all that apply) | ☐ Better digital tools ☐ More advanced integration into clinical systems ☐ More time during appointments ☐ Training on outcome measurement ☐ A simpler process ☐ Clinical Director and/or Practice Manager “buy-in” ☐ Not relevant ☐ I do not think I should be collecting outcomes more regularly ☐ Other |
