# Supplementary figures and images for: Patterns and predictors of outcome monitoring amongst link workers: Learnings from the National Social Prescribing Link Worker Survey 2025
Source: PLoS One. 2026 Apr 29;21(4):e0346234. doi: 10.1371/journal.pone.0346234 (PMC13127906; doi:10.1371/journal.pone.0346234)

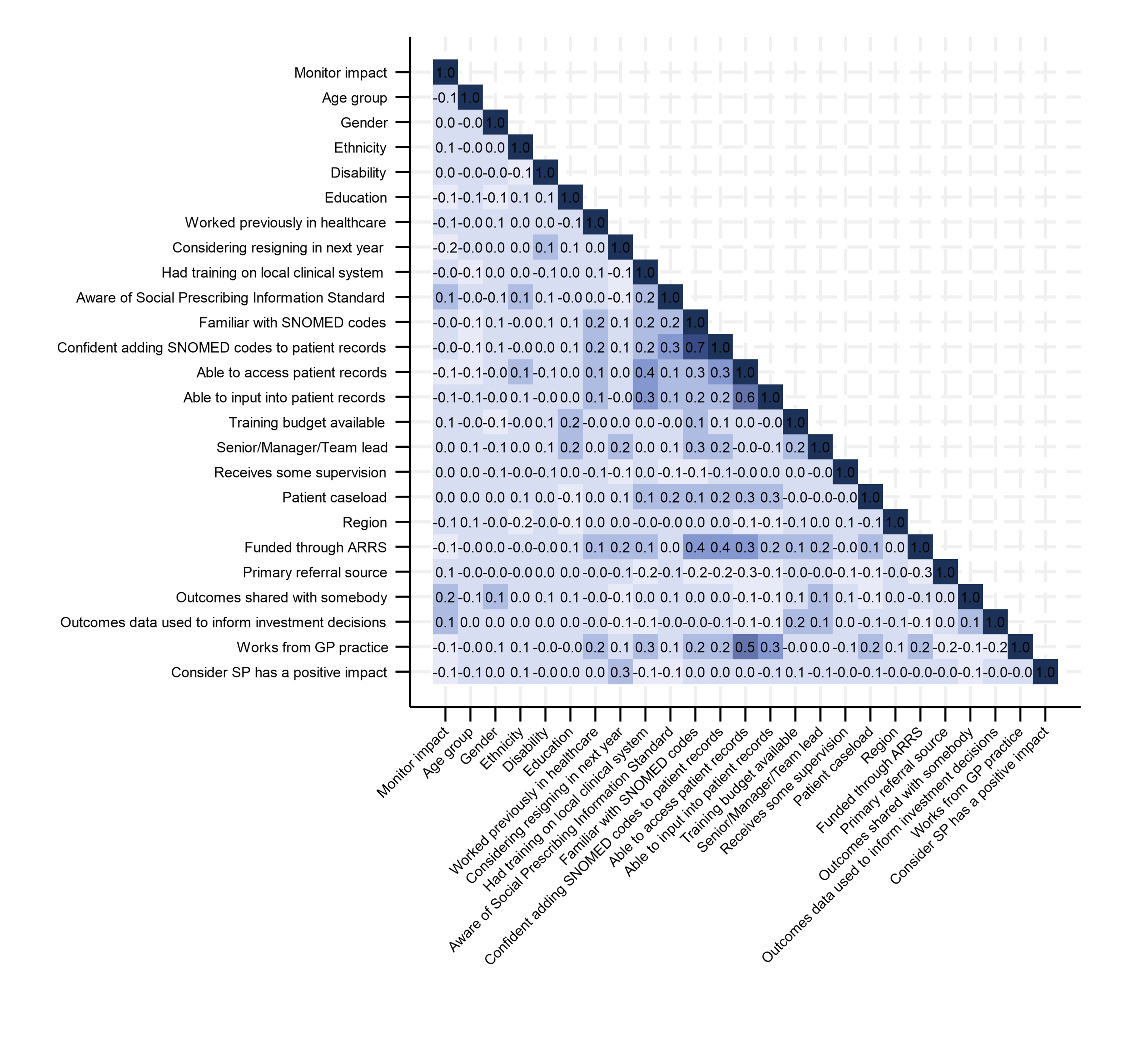

Supplement: S1 Fig — (PNG) [file pone.0346234.s002.png]

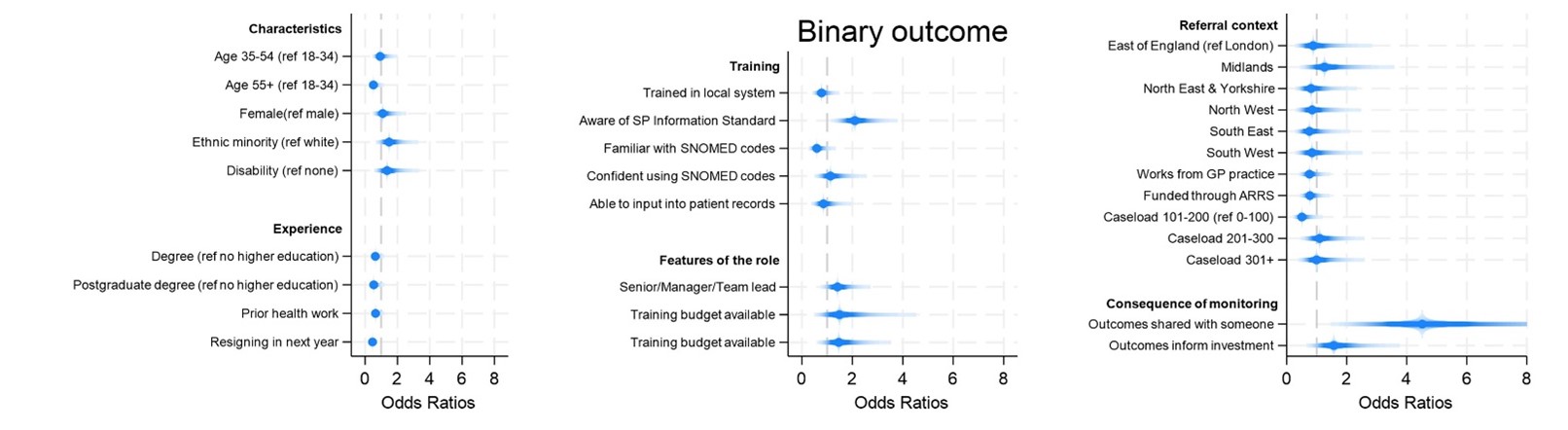

Supplement: S2 Fig — Notes: Ordinal outcomes consider which factors predict movement across any levels of the 5-point scale. Age and gender were included in all models. (JPG) [file pone.0346234.s003.jpg]

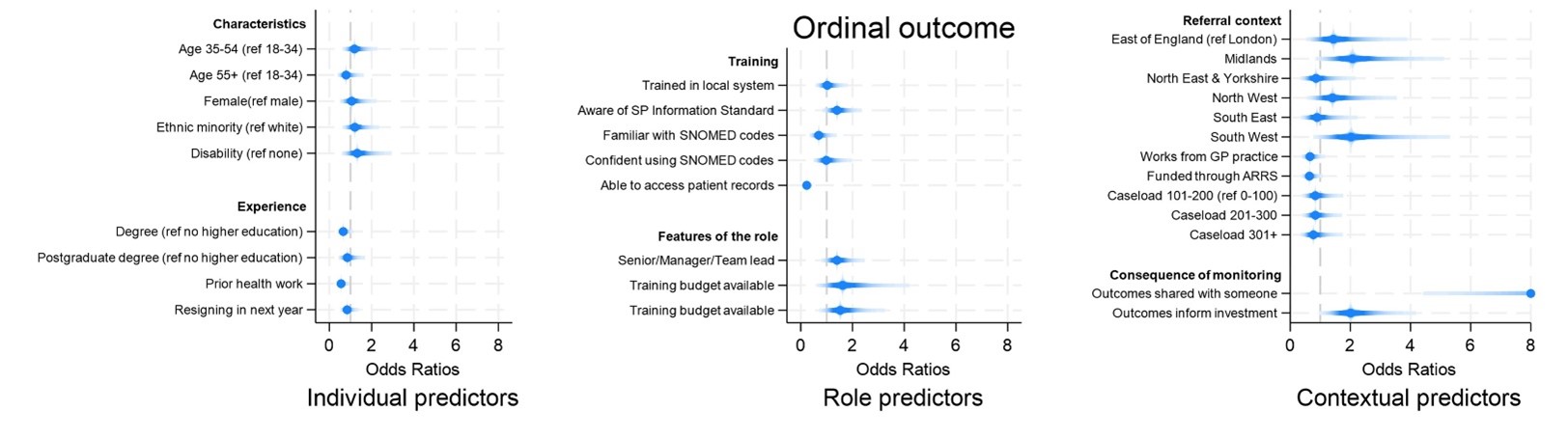

Supplement: S3 Fig — Notes: Ordinal outcomes consider which factors predict movement across any levels of the 5-point scale. Age and gender were included in all models. (JPG) [file pone.0346234.s004.jpg]
