# Supplementary material for: Patterns and predictors of outcome monitoring amongst link workers: Learnings from the National Social Prescribing Link Worker Survey 2025
Source: PLoS One. 2026 Apr 29;21(4):e0346234. doi: 10.1371/journal.pone.0346234 (PMC13127906; doi:10.1371/journal.pone.0346234)
Supplement: S1 Table — (DOCX) [file pone.0346234.s005.docx]

| **Supplementary Table 1: Sample Characteristics by impact monitoring** | | | | |  |
| --- | --- | --- | --- | --- | --- |
|  | Monitor impact | | | | |
|  | No | Yes | Total | Test | |
| N | 189 (46.2%) | 220 (53.8%) | 409 (100.0%) |  | |
| Age group |  |  |  |  | |
| 18-24 | 3 (1.6%) | 6 (2.8%) | 9 (2.3%) | 0.385 | |
| 25-34 | 29 (15.6%) | 33 (15.6%) | 62 (15.6%) |  | |
| 35-44 | 36 (19.4%) | 50 (23.7%) | 86 (21.7%) |  | |
| 45-54 | 55 (29.6%) | 69 (32.7%) | 124 (31.2%) |  | |
| 55-64 | 57 (30.6%) | 45 (21.3%) | 102 (25.7%) |  | |
| 65+ | 6 (3.2%) | 8 (3.8%) | 14 (3.5%) |  | |
| Gender |  |  |  |  | |
| Male | 24 (13.1%) | 26 (12.0%) | 50 (12.5%) | 0.733 | |
| Female | 159 (86.9%) | 191 (88.0%) | 350 (87.5%) |  | |
| Ethnicity |  |  |  |  | |
| White | 155 (87.1%) | 171 (83.0%) | 326 (84.9%) | 0.267 | |
| Other ethnic group | 23 (12.9%) | 35 (17.0%) | 58 (15.1%) |  | |
| Disability |  |  |  |  | |
| No | 169 (89.4%) | 194 (89.4%) | 363 (89.4%) | 0.996 | |
| Yes | 20 (10.6%) | 23 (10.6%) | 43 (10.6%) |  | |
| Education |  |  |  |  | |
| 1. Up to A-level/Btec nationals/advanced apprenticeship | 56 (29.6%) | 89 (40.5%) | 145 (35.5%) | 0.055 | |
| 2. Undergraduate degree/foundation degree/higher apprenticeship | 95 (50.3%) | 99 (45.0%) | 194 (47.4%) |  | |
| 3. Master's degree/PhD | 38 (20.1%) | 32 (14.5%) | 70 (17.1%) |  | |
| Job role |  |  |  |  | |
| 1. SPLW (Primary Care Network) | 112 (59.3%) | 101 (45.9%) | 213 (52.1%) | 0.059 | |
| 2. SPLW (VCFSE) | 37 (19.6%) | 67 (30.5%) | 104 (25.4%) |  | |
| 3. SPLW (GP Federation) | 22 (11.6%) | 22 (10.0%) | 44 (10.8%) |  | |
| 4. SPLW (Local Authority) | 13 (6.9%) | 16 (7.3%) | 29 (7.1%) |  | |
| 5. SPLW (NHS Trust) | 3 (1.6%) | 6 (2.7%) | 9 (2.2%) |  | |
| 6. SPLW (Housing Organisation) | 1 (0.5%) | 2 (0.9%) | 3 (0.7%) |  | |
| 7. Other | 1 (0.5%) | 6 (2.7%) | 7 (1.7%) |  | |
| Region |  |  |  |  | |
| 1. East of England | 20 (10.6%) | 21 (9.5%) | 41 (10.0%) | 0.548 | |
| 2. London | 26 (13.8%) | 32 (14.5%) | 58 (14.2%) |  | |
| 3. Midlands | 25 (13.2%) | 46 (20.9%) | 71 (17.4%) |  | |
| 4. North East & Yorkshire | 29 (15.3%) | 29 (13.2%) | 58 (14.2%) |  | |
| 5. North West | 29 (15.3%) | 34 (15.5%) | 63 (15.4%) |  | |
| 6. South East | 34 (18.0%) | 33 (15.0%) | 67 (16.4%) |  | |
| 7. South West | 26 (13.8%) | 25 (11.4%) | 51 (12.5%) |  | |
| Patient caseload |  |  |  |  | |
| 1. 0-100 | 27 (14.3%) | 45 (20.5%) | 72 (17.6%) | 0.052 | |
| 2. 101-200 | 62 (32.8%) | 47 (21.4%) | 109 (26.7%) |  | |
| 3. 201-300 | 62 (32.8%) | 81 (36.8%) | 143 (35.0%) |  | |
| 4. 301+ | 38 (20.1%) | 47 (21.4%) | 85 (20.8%) |  | |
| Primary referral source |  |  |  |  | |
| 1. GPs | 131 (69.3%) | 123 (55.9%) | 254 (62.1%) | 0.137 | |
| 2. PCN staff | 42 (22.2%) | 72 (32.7%) | 114 (27.9%) |  | |
| 3. self-referrals | 4 (2.1%) | 8 (3.6%) | 12 (2.9%) |  | |
| 4. Social services | 2 (1.1%) | 4 (1.8%) | 6 (1.5%) |  | |
| 5. Secondary care | 1 (0.5%) | 2 (0.9%) | 3 (0.7%) |  | |
| 6. Other (schools, CAMHS, VCFSE etc) | 9 (4.8%) | 11 (5.0%) | 20 (4.9%) |  | |
| Funded through ARRS |  |  |  |  | |
| No | 70 (37.0%) | 106 (48.2%) | 176 (43.0%) | 0.023 | |
| Yes | 119 (63.0%) | 114 (51.8%) | 233 (57.0%) |  | |
| Works from GP practice |  |  |  |  | |
| No | 61 (32.3%) | 96 (43.6%) | 157 (38.4%) | 0.018 | |
| Yes | 128 (67.7%) | 124 (56.4%) | 252 (61.6%) |  | |
| Receives some supervision |  |  |  |  | |
| No | 13 (6.9%) | 13 (5.9%) | 26 (6.4%) | 0.689 | |
| Yes | 176 (93.1%) | 207 (94.1%) | 383 (93.6%) |  | |
| Worked previously in healthcare |  |  |  |  | |
| No | 129 (68.3%) | 163 (74.1%) | 292 (71.4%) | 0.193 | |
| Yes | 60 (31.7%) | 57 (25.9%) | 117 (28.6%) |  | |
| N=409 | | | | |  |
