# Supplementary material for: Patterns and predictors of outcome monitoring amongst link workers: Learnings from the National Social Prescribing Link Worker Survey 2025
Source: PLoS One. 2026 Apr 29;21(4):e0346234. doi: 10.1371/journal.pone.0346234 (PMC13127906; doi:10.1371/journal.pone.0346234)
Supplement: S2 Table — (DOCX) [file pone.0346234.s006.docx]

| **Supplementary Table 2: Logistic regression model for monitoring outcomes often or very often; odds ratios and confidence intervals** | | | | | |  |
| --- | --- | --- | --- | --- | --- | --- |
|  |  |  | 1 | 2 | 3 | |
| Age | 35-54 | | 0.93 [0.52, 1.65] | 0.69 [0.50, 0.94] | 0.75 [0.55, 1.03] | |
|  | 55+ | | 0.51 [0.27, 0.97] |  |  |  |
| Gender | | | 1.09 [0.57, 2.09] | 1.41 [0.74, 2.70] | 1.01 [0.52, 1.96] | |
| Ethnicity | | | 1.48 [0.81, 2.72] |  |  | |
| Disability | | | 1.36 [0.68, 2.70] |  |  | |
| Education | 2. Undergraduate degree/foundation degree/higher apprenticeship | | 0.63 [0.39, 1.02] |  |  | |
|  | 3. Master's degree/PhD | | 0.54 [0.29, 0.99] |  |  | |
| Worked previously in healthcare | | | 0.65 [0.41, 1.03] |  |  | |
| Considering resigning in next year | | | 0.46 [0.29, 0.71] |  |  | |
| Had training on local clinical system | | |  | 0.78 [0.48, 1.27] |  | |
| Aware of Social Prescribing Information Standard | | |  | **2.10 [1.34, 3.30]** |  | |
| Familiar with SNOMED codes | | |  | 0.59 [0.32, 1.11] |  | |
| Confident adding SNOMED codes to patient records | | |  | 1.13 [0.61, 2.11] |  | |
| Able to input into patient records | | |  | 0.85 [0.45, 1.61] |  | |
| Senior/Manager/Team lead | | |  | 1.41 [0.85, 2.33] |  | |
| Receives some supervision | | |  | 1.49 [0.64, 3.47] |  | |
| Training budget available | | |  | 1.46 [0.75, 2.86] |  | |
| Region | 1. East of England | |  |  | 0.88 [0.36, 2.15] | |
|  | 3. Midlands | |  |  | 1.26 [0.57, 2.79] | |
|  | 4. North East & Yorkshire | |  |  | 0.81 [0.36, 1.81] | |
|  | 5. North West | |  |  | 0.85 [0.38, 1.91] | |
|  | 6. South East | |  |  | 0.76 [0.35, 1.65] | |
|  | 7. South West | |  |  | 0.84 [0.37, 1.94] | |
| Works from GP practice | | |  |  | 0.76 [0.47, 1.24] | |
| Funded through ARRS | | |  |  | 0.77 [0.49, 1.23] | |
| Patient caseload | 2. 101-200 | |  |  | 0.51 [0.26, 0.98] | |
|  | 3. 201-300 | |  |  | 1.10 [0.57, 2.11] | |
|  | 4. 301+ | |  |  | 0.99 [0.48, 2.07] | |
| Outcomes shared with somebody | | |  |  | **4.52 [1.92,10.63]** | |
| Outcomes data used to inform investment decisions | | |  |  | **1.57 [0.81, 3.05]** | |
| Intercept | | | 2.58 [1.08, 6.17] | 1.25 [0.32, 4.83] | 0.87 [0.22, 3.49] | |
| Number of observations | | | 377 | 377 | 377 | |
| *Reference categories: Age 18-34; Male; White; No disability; no higher education; London; caseload 0-100* | | | | | |  |
