# Supplementary material for: Patterns and predictors of outcome monitoring amongst link workers: Learnings from the National Social Prescribing Link Worker Survey 2025
Source: PLoS One. 2026 Apr 29;21(4):e0346234. doi: 10.1371/journal.pone.0346234 (PMC13127906; doi:10.1371/journal.pone.0346234)
Supplement: S3 Table — (DOCX) [file pone.0346234.s007.docx]

| **Supplementary Table 3: Ordinal regression model for monitoring outcomes; odds ratios and confidence intervals** | | | | | |
| --- | --- | --- | --- | --- | --- |
|  |  |  | 1 | 2 | 3 |
| Age | 35-54 | | 0.86 [0.52, 1.42] | 0.68 [0.51, 0.89] | 0.69 [0.52, 0.91] |
|  | 55+ | | 0.48 [0.27, 0.83] |  |  |
| Gender | | | 0.82 [0.47, 1.42] | 1.00 [0.58, 1.72] | 0.66 [0.38, 1.15] |
| Ethnicity | | | 1.87 [1.14, 3.07] |  |  |
| Disability | | | 1.24 [0.67, 2.32] |  |  |
| Education | 2. Undergraduate degree/foundation degree/higher apprenticeship | | 0.77 [0.51, 1.16] |  |  |
|  | 3. Master's degree/PhD | | 0.81 [0.48, 1.37] |  |  |
| Worked previously in healthcare | | | 0.67 [0.44, 1.00] |  |  |
| Considering resigning in next year | | | 0.56 [0.38, 0.81] |  |  |
| Had training on local clinical system | | |  | 1.00 [0.65, 1.53] |  |
| Aware of Social Prescribing Information Standard | | |  | **2.29 [1.53, 3.42]** |  |
| Familiar with SNOMED codes | | |  | 0.70 [0.41, 1.19] |  |
| Confident adding SNOMED codes to patient records | | |  | 1.11 [0.65, 1.87] |  |
| Able to input into patient records | | |  | 0.84 [0.48, 1.45] |  |
| Senior/Manager/Team lead | | |  | 1.43 [0.93, 2.19] |  |
| Receives some supervision | | |  | 1.44 [0.69, 3.00] |  |
| Training budget available | | |  | 1.28 [0.73, 2.25] |  |
| Region | 1. East of England | |  |  | 0.99 [0.46, 2.12] |
|  | 3. Midlands | |  |  | 0.83 [0.43, 1.61] |
|  | 4. North East & Yorkshire | |  |  | 0.53 [0.27, 1.03] |
|  | 5. North West | |  |  | 0.86 [0.44, 1.68] |
|  | 6. South East | |  |  | 0.51 [0.27, 0.99] |
|  | 7. South West | |  |  | 0.52 [0.25, 1.07] |
| Works from GP practice | | |  |  | 0.85 [0.56, 1.30] |
| Funded through ARRS | | |  |  | 0.75 [0.50, 1.11] |
| Patient caseload | 2. 101-200 | |  |  | 0.62 [0.35, 1.11] |
|  | 3. 201-300 | |  |  | 1.09 [0.63, 1.90] |
|  | 4. 301+ | |  |  | 1.24 [0.66, 2.32] |
| Outcomes shared with somebody | | |  |  | **6.03 [2.93,12.43]** |
| Outcomes data used to inform investment decisions | | |  |  | **1.76 [1.03, 3.02]** |
| Number of observations | | | 377 | 377 | 377 |
| *Reference categories: Age 18-34; Male; White; No disability; no higher education; London; caseload 0-100* | | | | | |
