# Supplementary material for: Patterns and predictors of outcome monitoring amongst link workers: Learnings from the National Social Prescribing Link Worker Survey 2025
Source: PLoS One. 2026 Apr 29;21(4):e0346234. doi: 10.1371/journal.pone.0346234 (PMC13127906; doi:10.1371/journal.pone.0346234)
Supplement: S4 Table — (DOCX) [file pone.0346234.s008.docx]

| **Supplementary Table 4: Logistic regression model for recording quantitative outcomes (e.g. ONS4) often or very often; odds ratios and confidence intervals** | | | | | |  |
| --- | --- | --- | --- | --- | --- | --- |
|  |  |  | 1 | 2 | 3 | |
| Age | 35-54 | | 1.38 [0.79, 2.42] | 0.84 [0.61, 1.16] | 0.97 [0.70, 1.34] | |
|  | 55+ | | 0.90 [0.48, 1.68] |  |  |  |
| Gender | | | 1.03 [0.54, 1.94] | 1.22 [0.63, 2.36] | 0.94 [0.47, 1.89] | |
| Ethnicity | | | 1.14 [0.64, 2.04] |  |  | |
| Disability | | | 1.38 [0.70, 2.70] |  |  | |
| Education | 2. Undergraduate degree/foundation degree/higher apprenticeship | | 0.65 [0.40, 1.03] |  |  | |
|  | 3. Master's degree/PhD | | 0.79 [0.43, 1.44] |  |  | |
| Worked previously in healthcare | | | 0.67 [0.42, 1.06] |  |  | |
| Considering resigning in next year | | | 0.85 [0.55, 1.30] |  |  | |
| Had training on local clinical system | | |  | 1.30 [0.76, 2.22] |  | |
| Aware of Social Prescribing Information Standard | | |  | 1.18 [0.75, 1.87] |  | |
| Familiar with SNOMED codes | | |  | 0.82 [0.43, 1.56] |  | |
| Confident adding SNOMED codes to patient records | | |  | 0.90 [0.47, 1.70] |  | |
| Able to input into patient records | | |  | **0.22 [0.11, 0.41]** |  | |
| Senior/Manager/Team lead | | |  | 1.26 [0.76, 2.10] |  | |
| Receives some supervision | | |  | 1.53 [0.63, 3.72] |  | |
| Training budget available | | |  | 1.57 [0.81, 3.08] |  | |
| Region | 1. East of England | |  |  | 2.04 [0.80, 5.17] | |
|  | 3. Midlands | |  |  | **2.37 [1.05, 5.36]** | |
|  | 4. North East & Yorkshire | |  |  | 1.09 [0.48, 2.49] | |
|  | 5. North West | |  |  | 1.71 [0.75, 3.93] | |
|  | 6. South East | |  |  | 1.12 [0.50, 2.52] | |
|  | 7. South West | |  |  | 1.93 [0.81, 4.61] | |
| Works from GP practice | | |  |  | 0.76 [0.47, 1.24] | |
| Funded through ARRS | | |  |  | 0.77 [0.49, 1.23] | |
| Patient caseload | 2. 101-200 | |  |  | 0.86 [0.44, 1.70] | |
|  | 3. 201-300 | |  |  | 0.86 [0.44, 1.68] | |
|  | 4. 301+ | |  |  | 0.70 [0.33, 1.47] | |
| Outcomes shared with somebody | | |  |  | **7.17 [2.36,21.76]** | |
| Outcomes data used to inform investment decisions | | |  |  | **2.59 [1.29, 5.22]** | |
| Number of observations | | | 377 | 377 | 377 | |
| *Reference categories: Age 18-34; Male; White; No disability; no higher education; London; caseload 0-100* | | | | | |  |
