# Supplementary material for: Patterns and predictors of outcome monitoring amongst link workers: Learnings from the National Social Prescribing Link Worker Survey 2025
Source: PLoS One. 2026 Apr 29;21(4):e0346234. doi: 10.1371/journal.pone.0346234 (PMC13127906; doi:10.1371/journal.pone.0346234)
Supplement: S5 Table — (DOCX) [file pone.0346234.s009.docx]

| **Supplementary Table 5: Ordinal regression model for recording quantitative outcomes (e.g. ONS4); odds ratios and confidence intervals** | | | | | |
| --- | --- | --- | --- | --- | --- |
|  |  |  | 1 | 2 | 3 |
| Age | 35-54 | | 1.19 [0.73, 1.94] | 0.78 [0.59, 1.02] | 0.89 [0.67, 1.18] |
|  | 55+ | | 0.79 [0.46, 1.36] |  |  |
| Gender | | | 1.06 [0.60, 1.87] | 1.27 [0.71, 2.26] | 0.87 [0.48, 1.57] |
| Ethnicity | | | 1.21 [0.73, 2.00] |  |  |
| Disability | | | 1.32 [0.71, 2.43] |  |  |
| Education | 2. Undergraduate degree/foundation degree/higher apprenticeship | | 0.66 [0.43, 0.99] |  |  |
|  | 3. Master's degree/PhD | | 0.85 [0.51, 1.43] |  |  |
| Worked previously in healthcare | | | 0.55 [0.37, 0.84] |  |  |
| Considering resigning in next year | | | 0.84 [0.58, 1.22] |  |  |
| Had training on local clinical system | | |  | 1.02 [0.66, 1.59] |  |
| Aware of Social Prescribing Information Standard | | |  | 1.40 [0.95, 2.08] |  |
| Familiar with SNOMED codes | | |  | 0.69 [0.41, 1.18] |  |
| Confident adding SNOMED codes to patient records | | |  | 0.98 [0.58, 1.67] |  |
| Able to input into patient records | | |  | **0.23 [0.14, 0.39]** |  |
| Senior/Manager/Team lead | | |  | 1.40 [0.91, 2.16] |  |
| Receives some supervision | | |  | 1.62 [0.79, 3.34] |  |
| Training budget available | | |  | 1.53 [0.86, 2.72] |  |
| Region | 1. East of England | |  |  | 1.44 [0.67, 3.07] |
|  | 3. Midlands | |  |  | **2.07 [1.04, 4.12]** |
|  | 4. North East & Yorkshire | |  |  | 0.86 [0.42, 1.74] |
|  | 5. North West | |  |  | 1.41 [0.70, 2.84] |
|  | 6. South East | |  |  | 0.89 [0.44, 1.79] |
|  | 7. South West | |  |  | 2.02 [0.97, 4.21] |
| Works from GP practice | | |  |  | 0.85 [0.56, 1.30] |
| Funded through ARRS | | |  |  | 0.75 [0.50, 1.11] |
| Patient caseload | 2. 101-200 | |  |  | 0.83 [0.47, 1.47] |
|  | 3. 201-300 | |  |  | 0.84 [0.48, 1.45] |
|  | 4. 301+ | |  |  | 0.77 [0.41, 1.43] |
| Outcomes shared with somebody | | |  |  | **13.58 [5.78,31.90]** |
| Outcomes data used to inform investment decisions | | |  |  | **2.01 [1.15, 3.51]** |
| Number of observations | | | 377 | 377 | 377 |
| *Reference categories: Age 18-34; Male; White; No disability; no higher education; London; caseload 0-100* | | | | | |
