# Supplementary material for: Patterns and predictors of outcome monitoring amongst link workers: Learnings from the National Social Prescribing Link Worker Survey 2025
Source: PLoS One. 2026 Apr 29;21(4):e0346234. doi: 10.1371/journal.pone.0346234 (PMC13127906; doi:10.1371/journal.pone.0346234)
Supplement: S6 Table — (DOCX) [file pone.0346234.s010.docx]

| **Supplementary Table 6: Perceived impacts by impact monitoring/reporting** | | | |
| --- | --- | --- | --- |
|  | Not monitoring | Monitoring | Total |
| N | 189 (46.2%) | 220 (53.8%) | 409 (100.0%) |
| Consider SP has a positive impact |  |  |  |
| 1. Strongly agree | 123 (65.1%) | 166 (75.5%) | 289 (70.7%) |
| 2. Agree | 58 (30.7%) | 53 (24.1%) | 111 (27.1%) |
| 3. Neither agree nor disagree | 6 (3.2%) | 1 (0.5%) | 7 (1.7%) |
| 4. Disagree | 1 (0.5%) | 0 (0.0%) | 1 (0.2%) |
| 5. Strongly disagree | 1 (0.5%) | 0 (0.0%) | 1 (0.2%) |
| PERCEIVED IMPACT ON...  Physical health | | | |
| 2. Positive | 32 (50.0%) | 81 (87.1%) | 113 (72.0%) |
| 3. No change | 14 (21.9%) | 5 (5.4%) | 19 (12.1%) |
| 4. Negative | 18 (28.1%) | 7 (7.5%) | 25 (15.9%) |
| Mental health |  |  |  |
| 2. Positive | 66 (82.5%) | 106 (95.5%) | 172 (90.1%) |
| 3. No change | 4 (5.0%) | 0 (0.0%) | 4 (2.1%) |
| 4. Negative | 10 (12.5%) | 5 (4.5%) | 15 (7.9%) |
| Social connection |  |  |  |
| 2. Positive | 105 (93.8%) | 154 (97.5%) | 259 (95.9%) |
| 3. No change | 3 (2.7%) | 0 (0.0%) | 3 (1.1%) |
| 4. Negative | 4 (3.6%) | 4 (2.5%) | 8 (3.0%) |
| GP contacts |  |  |  |
| 2. Positive | 115 (70.1%) | 122 (59.5%) | 237 (64.2%) |
| 3. No change | 12 (7.3%) | 18 (8.8%) | 30 (8.1%) |
| 4. Negative | 0 (0.0%) | 1 (0.5%) | 1 (0.3%) |
| 5. Strong negative | 37 (22.6%) | 64 (31.2%) | 101 (27.4%) |
| Hospital contacts |  |  |  |
| 1. Strong positive | 16 (12.3%) | 33 (18.4%) | 49 (15.9%) |
| 2. Positive | 79 (60.8%) | 107 (59.8%) | 186 (60.2%) |
| 3. No change | 35 (26.9%) | 37 (20.7%) | 72 (23.3%) |
| 4. Negative | 0 (0.0%) | 1 (0.6%) | 1 (0.3%) |
| 5. Strong negative | 0 (0.0%) | 1 (0.6%) | 1 (0.3%) |
| Number of medications |  |  |  |
| 1. Strong positive | 11 (9.7%) | 27 (16.1%) | 38 (13.5%) |
| 2. Positive | 52 (46.0%) | 84 (50.0%) | 136 (48.4%) |
| 3. No change | 49 (43.4%) | 55 (32.7%) | 104 (37.0%) |
| 4. Negative | 0 (0.0%) | 2 (1.2%) | 2 (0.7%) |
| 5. Strong negative | 1 (0.9%) | 0 (0.0%) | 1 (0.4%) |
| Ability to work |  |  |  |
| 1. Strong positive | 13 (8.9%) | 42 (22.6%) | 55 (16.6%) |
| 2. Positive | 101 (69.2%) | 117 (62.9%) | 218 (65.7%) |
| 3. No change | 31 (21.2%) | 25 (13.4%) | 56 (16.9%) |
| 4. Negative | 0 (0.0%) | 1 (0.5%) | 1 (0.3%) |
| 5. Strong negative | 1 (0.7%) | 1 (0.5%) | 2 (0.6%) |

*Note: These differences were not tested statistically or adjusted for demographics.*
