# Supplementary material for: Patterns and predictors of outcome monitoring amongst link workers: Learnings from the National Social Prescribing Link Worker Survey 2025
Source: PLoS One. 2026 Apr 29;21(4):e0346234. doi: 10.1371/journal.pone.0346234 (PMC13127906; doi:10.1371/journal.pone.0346234)
Supplement: S7 Table — (DOCX) [file pone.0346234.s011.docx]

| **Supplementary Table 7: Perceived impacts by use of patient outcome measures** | | | |
| --- | --- | --- | --- |
|  | Using | Not using | Total |
| N | 210 (51.3%) | 199 (48.7%) | 409 (100.0%) |
| Consider SP has a positive impact |  |  |  |
| 1. Strongly agree | 137 (65.2%) | 152 (76.4%) | 289 (70.7%) |
| 2. Agree | 67 (31.9%) | 44 (22.1%) | 111 (27.1%) |
| 3. Neither agree nor disagree | 5 (2.4%) | 2 (1.0%) | 7 (1.7%) |
| 4. Disagree | 0 (0.0%) | 1 (0.5%) | 1 (0.2%) |
| 5. Strongly disagree | 1 (0.5%) | 0 (0.0%) | 1 (0.2%) |
| PERCEIVED IMPACT ON...  Physical health | | | |
| 2. Positive | 47 (61.8%) | 66 (81.5%) | 113 (72.0%) |
| 3. No change | 11 (14.5%) | 8 (9.9%) | 19 (12.1%) |
| 4. Negative | 18 (23.7%) | 7 (8.6%) | 25 (15.9%) |
| Mental health |  |  |  |
| 2. Positive | 84 (88.4%) | 88 (91.7%) | 172 (90.1%) |
| 3. No change | 1 (1.1%) | 3 (3.1%) | 4 (2.1%) |
| 4. Negative | 10 (10.5%) | 5 (5.2%) | 15 (7.9%) |
| Social connection |  |  |  |
| 2. Positive | 133 (97.1%) | 126 (94.7%) | 259 (95.9%) |
| 3. No change | 1 (0.7%) | 2 (1.5%) | 3 (1.1%) |
| 4. Negative | 3 (2.2%) | 5 (3.8%) | 8 (3.0%) |
| GP contacts |  |  |  |
| 2. Positive | 122 (64.9%) | 115 (63.5%) | 237 (64.2%) |
| 3. No change | 14 (7.4%) | 16 (8.8%) | 30 (8.1%) |
| 4. Negative | 0 (0.0%) | 1 (0.6%) | 1 (0.3%) |
| 5. Strong negative | 52 (27.7%) | 49 (27.1%) | 101 (27.4%) |
| Hospital contacts |  |  |  |
| 1. Strong positive | 23 (14.4%) | 26 (17.4%) | 49 (15.9%) |
| 2. Positive | 101 (63.1%) | 85 (57.0%) | 186 (60.2%) |
| 3. No change | 36 (22.5%) | 36 (24.2%) | 72 (23.3%) |
| 4. Negative | 0 (0.0%) | 1 (0.7%) | 1 (0.3%) |
| 5. Strong negative | 0 (0.0%) | 1 (0.7%) | 1 (0.3%) |
| Number of medications |  |  |  |
| 1. Strong positive | 16 (11.7%) | 22 (15.3%) | 38 (13.5%) |
| 2. Positive | 62 (45.3%) | 74 (51.4%) | 136 (48.4%) |
| 3. No change | 58 (42.3%) | 46 (31.9%) | 104 (37.0%) |
| 4. Negative | 1 (0.7%) | 1 (0.7%) | 2 (0.7%) |
| 5. Strong negative | 0 (0.0%) | 1 (0.7%) | 1 (0.4%) |
| Ability to work |  |  |  |
| 1. Strong positive | 21 (12.7%) | 34 (20.4%) | 55 (16.6%) |
| 2. Positive | 112 (67.9%) | 106 (63.5%) | 218 (65.7%) |
| 3. No change | 29 (17.6%) | 27 (16.2%) | 56 (16.9%) |
| 4. Negative | 1 (0.6%) | 0 (0.0%) | 1 (0.3%) |
| 5. Strong negative | 2 (1.2%) | 0 (0.0%) | 2 (0.6%) |

*Note: These differences were not tested statistically or adjusted for demographics.*
