# Supplementary material for: Patterns and predictors of outcome monitoring amongst link workers: Learnings from the National Social Prescribing Link Worker Survey 2025
Source: PLoS One. 2026 Apr 29;21(4):e0346234. doi: 10.1371/journal.pone.0346234 (PMC13127906; doi:10.1371/journal.pone.0346234)
Supplement: S8 Table — (DOCX) [file pone.0346234.s012.docx]

| **Supplementary Table 8: Perceived effectiveness of different interventions by impact monitoring/reporting** | | | |  |
| --- | --- | --- | --- | --- |
|  | Not monitoring | Monitoring | Total | |
| N | 189 (46.2%) | 220 (53.8%) | 409 (100.0%) | |
| Information & advice services | | | |  |
| 1. Very effective | 79 (42.2%) | 124 (57.7%) | 203 (50.5%) | |
| 2. Quite effective | 85 (45.5%) | 66 (30.7%) | 151 (37.6%) | |
| 3. Not very effective | 20 (10.7%) | 19 (8.8%) | 39 (9.7%) | |
| 4. Not at all effective | 3 (1.6%) | 6 (2.8%) | 9 (2.2%) | |
| Arts & cultural activities |  |  |  | |
| 1. Very effective | 41 (23.8%) | 58 (28.6%) | 99 (26.4%) | |
| 2. Quite effective | 96 (55.8%) | 109 (53.7%) | 205 (54.7%) | |
| 3. Not very effective | 29 (16.9%) | 31 (15.3%) | 60 (16.0%) | |
| 4. Not at all effective | 6 (3.5%) | 5 (2.5%) | 11 (2.9%) | |
| Heritage activities |  |  |  | |
| 1. Very effective | 9 (6.9%) | 21 (13.0%) | 30 (10.3%) | |
| 2. Quite effective | 64 (49.2%) | 86 (53.1%) | 150 (51.4%) | |
| 3. Not very effective | 45 (34.6%) | 48 (29.6%) | 93 (31.8%) | |
| 4. Not at all effective | 12 (9.2%) | 7 (4.3%) | 19 (6.5%) | |
| Nature-based activities |  |  |  | |
| 1. Very effective | 50 (28.6%) | 78 (38.6%) | 128 (34.0%) | |
| 2. Quite effective | 91 (52.0%) | 101 (50.0%) | 192 (50.9%) | |
| 3. Not very effective | 30 (17.1%) | 19 (9.4%) | 49 (13.0%) | |
| 4. Not at all effective | 4 (2.3%) | 4 (2.0%) | 8 (2.1%) | |
| Physical activities |  |  |  | |
| 1. Very effective | 66 (35.9%) | 122 (56.0%) | 188 (46.8%) | |
| 2. Quite effective | 102 (55.4%) | 85 (39.0%) | 187 (46.5%) | |
| 3. Not very effective | 14 (7.6%) | 9 (4.1%) | 23 (5.7%) | |
| 4. Not at all effective | 2 (1.1%) | 2 (0.9%) | 4 (1.0%) | |
| Age-related activities |  |  |  | |
| 1. Very effective | 71 (38.0%) | 102 (47.7%) | 173 (43.1%) | |
| 2. Quite effective | 98 (52.4%) | 97 (45.3%) | 195 (48.6%) | |
| 3. Not very effective | 16 (8.6%) | 13 (6.1%) | 29 (7.2%) | |
| 4. Not at all effective | 2 (1.1%) | 2 (0.9%) | 4 (1.0%) | |
| Faith-based organisations |  |  |  | |
| 1. Very effective | 25 (18.5%) | 38 (21.3%) | 63 (20.1%) | |
| 2. Quite effective | 78 (57.8%) | 100 (56.2%) | 178 (56.9%) | |
| 3. Not very effective | 24 (17.8%) | 38 (21.3%) | 62 (19.8%) | |
| 4. Not at all effective | 8 (5.9%) | 2 (1.1%) | 10 (3.2%) | |
| Healthcare services |  |  |  | |
| 1. Very effective | 40 (21.4%) | 83 (38.4%) | 123 (30.5%) | |
| 2. Quite effective | 107 (57.2%) | 101 (46.8%) | 208 (51.6%) | |
| 3. Not very effective | 37 (19.8%) | 28 (13.0%) | 65 (16.1%) | |
| 4. Not at all effective | 3 (1.6%) | 4 (1.9%) | 7 (1.7%) | |
| *Physical health (e.g. managing long-term conditions, mobility, Body Mass Index)*  *Mental health (e.g. anxiety, depression, overall wellbeing)*  *Social connection (e.g. loneliness, engagement with community)*  *GP contacts (e.g. frequency of visits, dependence on GP)*  *Hospital contacts (e.g. A&E visits, admissions)*  *Number of medications used Ability to work (e.g. time off sick, returning to work readiness)*  *Note: These differences were not tested statistically or adjusted for demographics.* | | | |  |
