# Supplementary material for: Patterns and predictors of outcome monitoring amongst link workers: Learnings from the National Social Prescribing Link Worker Survey 2025
Source: PLoS One. 2026 Apr 29;21(4):e0346234. doi: 10.1371/journal.pone.0346234 (PMC13127906; doi:10.1371/journal.pone.0346234)
Supplement: S9 Table — (DOCX) [file pone.0346234.s013.docx]

| **Supplementary Table 9: Perceived effectiveness of different interventions by use of patient outcome measures** | | | |  |
| --- | --- | --- | --- | --- |
|  | Not monitoring | Monitoring | Total | |
| N | 210 (51.3%) | 199 (48.7%) | 409 (100.0%) | |
| Information & advice services | | | |  |
| 1. Very effective | 99 (48.1%) | 104 (53.1%) | 203 (50.5%) | |
| 2. Quite effective | 77 (37.4%) | 74 (37.8%) | 151 (37.6%) | |
| 3. Not very effective | 27 (13.1%) | 12 (6.1%) | 39 (9.7%) | |
| 4. Not at all effective | 3 (1.5%) | 6 (3.1%) | 9 (2.2%) | |
| Arts & cultural activities |  |  |  | |
| 1. Very effective | 42 (22.5%) | 57 (30.3%) | 99 (26.4%) | |
| 2. Quite effective | 104 (55.6%) | 101 (53.7%) | 205 (54.7%) | |
| 3. Not very effective | 35 (18.7%) | 25 (13.3%) | 60 (16.0%) | |
| 4. Not at all effective | 6 (3.2%) | 5 (2.7%) | 11 (2.9%) | |
| Heritage activities |  |  |  | |
| 1. Very effective | 11 (7.5%) | 19 (13.0%) | 30 (10.3%) | |
| 2. Quite effective | 68 (46.6%) | 82 (56.2%) | 150 (51.4%) | |
| 3. Not very effective | 55 (37.7%) | 38 (26.0%) | 93 (31.8%) | |
| 4. Not at all effective | 12 (8.2%) | 7 (4.8%) | 19 (6.5%) | |
| Nature-based activities |  |  |  | |
| 1. Very effective | 52 (27.2%) | 76 (40.9%) | 128 (34.0%) | |
| 2. Quite effective | 102 (53.4%) | 90 (48.4%) | 192 (50.9%) | |
| 3. Not very effective | 33 (17.3%) | 16 (8.6%) | 49 (13.0%) | |
| 4. Not at all effective | 4 (2.1%) | 4 (2.2%) | 8 (2.1%) | |
| Physical activities |  |  |  | |
| 1. Very effective | 83 (40.5%) | 105 (53.3%) | 188 (46.8%) | |
| 2. Quite effective | 106 (51.7%) | 81 (41.1%) | 187 (46.5%) | |
| 3. Not very effective | 13 (6.3%) | 10 (5.1%) | 23 (5.7%) | |
| 4. Not at all effective | 3 (1.5%) | 1 (0.5%) | 4 (1.0%) | |
| Age-related activities |  |  |  | |
| 1. Very effective | 84 (41.2%) | 89 (45.2%) | 173 (43.1%) | |
| 2. Quite effective | 105 (51.5%) | 90 (45.7%) | 195 (48.6%) | |
| 3. Not very effective | 13 (6.4%) | 16 (8.1%) | 29 (7.2%) | |
| 4. Not at all effective | 2 (1.0%) | 2 (1.0%) | 4 (1.0%) | |
| Faith-based organisations |  |  |  | |
| 1. Very effective | 27 (17.2%) | 36 (23.1%) | 63 (20.1%) | |
| 2. Quite effective | 87 (55.4%) | 91 (58.3%) | 178 (56.9%) | |
| 3. Not very effective | 35 (22.3%) | 27 (17.3%) | 62 (19.8%) | |
| 4. Not at all effective | 8 (5.1%) | 2 (1.3%) | 10 (3.2%) | |
| Healthcare services |  |  |  | |
| 1. Very effective | 59 (28.6%) | 64 (32.5%) | 123 (30.5%) | |
| 2. Quite effective | 104 (50.5%) | 104 (52.8%) | 208 (51.6%) | |
| 3. Not very effective | 39 (18.9%) | 26 (13.2%) | 65 (16.1%) | |
| 4. Not at all effective | 4 (1.9%) | 3 (1.5%) | 7 (1.7%) | |
| *Physical health (e.g. managing long-term conditions, mobility, Body Mass Index)*  *Mental health (e.g. anxiety, depression, overall wellbeing)*  *Social connection (e.g. loneliness, engagement with community)*  *GP contacts (e.g. frequency of visits, dependence on GP)*  *Hospital contacts (e.g. A&E visits, admissions)*  *Number of medications used Ability to work (e.g. time off sick, returning to work readiness)*  *Note: These differences were not tested statistically or adjusted for demographics.* | | | |  |
